# Application of a portable sealed positive pressure infusion device in a porcine model of hemorrhagic shock

**DOI:** 10.3389/fmed.2026.1738724

**Published:** 2026-01-15

**Authors:** Jinsong Tao, Yuandan Lai, Caiyuan Fu, Yiru Lin, Yuemin Zhang, Wenting Huang, Zhicheng Huang, Chunlan Tian, Weihang Wu, Meijiao Lin

**Affiliations:** 1 Department of General Surgery, Fuzong Teaching Hospital of Fujian University of Traditional Chinese Medicine (900th Hospital), Fuzhou, Fujian, China; 2 Department of General Surgery, 900th Hospital of PLA Joint Logistic Support Force, Fuzhou, Fujian, China; 3 Nursing College, Fujian University of Traditional Chinese Medicine, Fuzhou, Fujian, China; 4 Fuzong Clinical Medical College of Fujian Medical University, 900th Hospital of PLA Joint Logistic Support Force, Fuzhou, Fujian, China

**Keywords:** fluid resuscitation, hemorrhagic shock, porcine model, portable infusion device, prehospital care

## Abstract

**Background:**

Hemorrhagic shock is a leading cause of trauma-related mortality in military and disaster settings, necessitating prompt fluid resuscitation. Conventional infusion methods, which rely on elevating fluid containers to generate hydrostatic pressure, risk secondary injury and are less efficient during prehospital transport. We developed a wearable, portable, sealed positive-pressure infusion device to optimize fluid administration in austere environments.

**Methods:**

Ten male Bama miniature pigs underwent controlled hemorrhagic shock (mean arterial pressure [MAP] < 60 mmHg, induced by blood withdrawal until approximately 40–45% of estimated total blood volume [70 mL/kg body weight] and maintained for 30 min). Animals were randomized to receive resuscitation with 6% hydroxyethyl starch 130/0.4 in saline at 10 mL/min using either the novel device or conventional manual pressure infusion. Vital signs and blood parameters (e.g., activated partial thromboplastin time [APTT]) were measured at baseline, shock phase, 1 h post-resuscitation, and upon completion of resuscitation. Resuscitation time, fluid volume, and maximum infusion line pressure were recorded.

**Results:**

All animals achieved successful resuscitation (100% immediate survival). During a 7-day follow-up, one animal in each group died (20%, *p* > 0.05). The device group exhibited a significantly shorter resuscitation time (103.0 ± 16.97 [95% CI: 81.93, 124.07] vs. 122.4 ± 8.02 [95% CI: 112.44, 132.36] minutes, *p* = 0.0496) and a higher infusion rate (9.29 ± 0.50 [95% CI: 8.67, 9.91] vs. 7.68 ± 0.40 [95% CI: 7.18, 8.18] mL/min, *p* < 0.001) compared with the control group. Both groups exhibited reduced APTT post-resuscitation; these descriptive findings may be associated with the colloid used but require further investigation with comprehensive coagulation assays (e.g., viscoelastic testing) and do not imply clinical benefit or harm.

**Conclusion:**

The portable sealed positive-pressure infusion device provided stable pressure, improved infusion performance, and maintained safety. It represents a feasible solution for managing hemorrhagic shock in prehospital austere settings.

## 1 Introduction

Hemorrhagic shock is a critical condition characterized by a rapid reduction in circulating blood volume, impaired tissue perfusion, and cellular metabolic dysfunction due to massive blood loss. It is a leading cause of mortality among young and middle-aged adults, accounting for 30–40% of trauma-related deaths worldwide (1), and is particularly severe in disaster and war settings, where more than 80% of casualties succumb to early hemorrhagic shock (2). Prompt fluid resuscitation remains the most common intervention for managing hemorrhagic shock (3–5). Studies have shown that initiating emergency treatment within 30 min of injury and completing fluid resuscitation within 1 h can reduce mortality by two-thirds, underscoring an inverse relationship between the treatment time window and mortality rates (6–8).

However, disaster sites often pose environmental and logistical challenges, including complex terrain, limited treatment space, and shortages of personnel and resources. Consequently, approximately 27–58% of casualties succumb during the prehospital phase due to delayed trauma care (9, 10). In complex environments, equipment portability affects resuscitation efficiency, and studies recommend investing in compact devices (11–13).

Currently, prehospital fluid resuscitation typically relies on gravity-driven and manual pressure infusion devices, which exhibit significant limitations in disaster areas (14). Traditional gravity-driven devices depend on elevating the infusion container to create a hydrostatic pressure gradient based on height differences for fluid delivery. This setup is prone to instability in dynamic environments, increasing risks of secondary injury, air embolism, and casualty exposure in battlefield settings. Maintaining these devices requires a substantial amount of personnel, posing safety hazards. Manual pressure devices deliver fluids using pressure cuffs or inflated airbags, independent of patient position. However, manual squeezing increases personnel demands, impedes precise pressure regulation, and risks vascular endothelium damage from excessive pressure. Electric pressure pumps reduce personnel needs but depend on power supplies, limiting applicability in disasters.

Although previous studies have developed portable infusion devices that are gravity-independent and battery-free for wartime use (15), their application in complex prehospital environments remains limited. These devices rely on high-tension torsion spring mechanisms for pressurization, with limitations including restricted functionality, inadequate pressure stability, and exposed infusion bags lacking protection. Fluctuating pressure may cause variable infusion rates, complicating maintenance of target blood pressure. This may lead to unstable tissue perfusion and hinder monitoring. Moreover, sudden excessive pressure may disrupt initial hemostatic clots, causing recurrent bleeding and exacerbating complications such as dilutional coagulopathy and hypothermia (16, 17).

Therefore, developing an infusion device that provides constant pressure, is portable and lightweight, operates independently of electrical power, adapts to diverse terrains, and requires minimal manual labor is essential for enhancing medical care efficiency in disaster settings. In our preliminary work, we developed a sealed positive pressure infusion device that uses an electric air pump for pressurization. Through pressure regulation and sensor feedback, it automatically maintains a constant air-cushion pressure (18). The device’s efficacy and safety were evaluated in the Bama miniature pig hemorrhagic shock model, an internationally recognized trauma model with physiological characteristics resembling those of humans. This study addresses challenges in prehospital intravenous therapy within complex environments, such as war zones and natural disasters.

## 2 Materials and methods

### 2.1 Regulation and approval

This study was approved by the Medical Ethics Committee of the 900th Hospital of the Joint Logistics Support Force of the Chinese People’s Liberation Army (protocol number 2024–21). All procedures were performed in accordance with the guidelines of the Experimental Animal Ethics Committee of the People’s Republic of China on animal protection and relevant national regulations, adhering to the ARRIVE guidelines for reporting animal experiments.

### 2.2 Description of homemade portable sealed positive pressure infusion device

**Figure 1:**
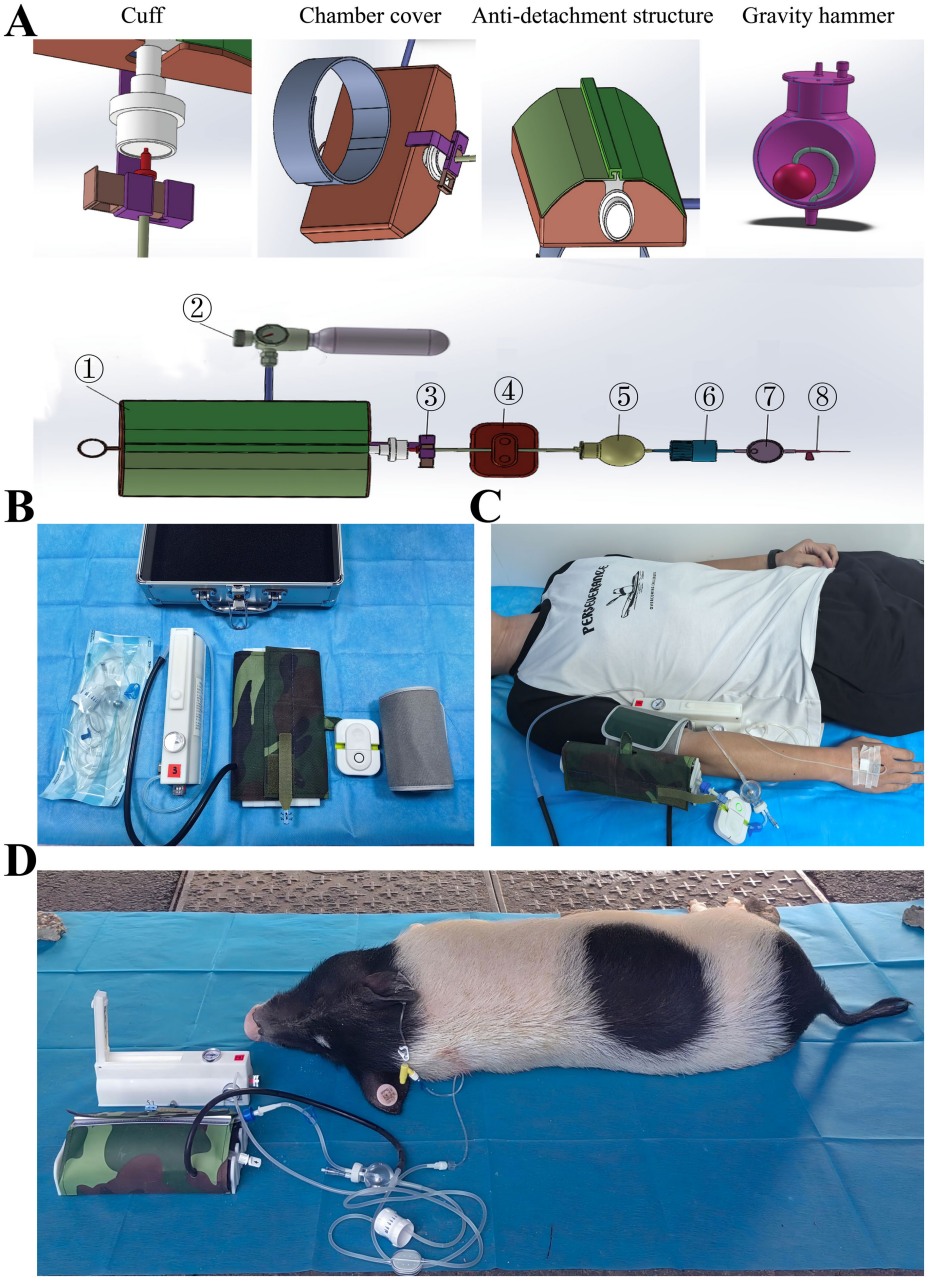
Design and application of the portable closed positive-pressure infusion device. **(A)** Schematic of device components: ① pressure chamber, ② pressure generator, ③ anti-detachment clamp, ④ alarm flow-arrest valve, ⑤ gravity exhaust bottle, ⑥ flow regulator, ⑦ filter, ⑧ needle. **(B)** Photograph of the actual device. **(C)** Device attached to the forelimb. **(D)** Device in use within a confined space.

The homemade portable sealed positive-pressure infusion device consists of three main components: a pressure generator, a pressure chamber, and an infusion system. The entire device is encased in aramid fiber fabric, providing a lightweight structure with excellent wear resistance and flame-retardant properties. The pressure chamber, a key component, measures 260 × 45 × 62 mm, while the pressure generator measures 217 × 10 × 4.3 mm. The overall device has a volume of approximately 0.0016 m^3^ and a mass of about 0.98 kg (Figure 1). A comparison of key features between the portable sealed positive-pressure infusion device and the conventional Clear-Cuff pressure infuser bag is provided in Supplementary Table 1.

### 2.3 Construction and working principle

The pressure generator consists of a compressed air cylinder and a pressure-reducing regulator. The output pressure is regulated via a precision valve core, with an adjustable range of 0–40 kPa. Infusion of 500 mL of liquid consumes 2.5 g of gas, enabling a standard 90 g cylinder to support up to 36 bags of 500 mL infusion. The pressure chamber consists of a flexible polymer body with Velcro cuffs and incorporates an anti-disengagement mechanism in the chamber lid. It is designed to accommodate infusion or blood transfusion bags with capacities ranging from 100 to 500 mL.

The infusion system consists of an alarm-equipped stopcock, a gravity-venting bottle, a flow regulator, and a 15-μm precision filter. All components comply with the standards specified in “Gravity-Fed Disposable Infusion Set” (GB 8368–2018), “Disposable Intravenous Infusion Needle” (GB 18671–2009), and “Test Methods for Infusion, Transfusion, and Injection Equipment for Medical Use - Part 1: Chemical Analysis Methods” (GB/T 14233.1–2008).

### 2.4 Operation and field application

The device is operated through a simple four-step procedure: (1) inserting the infusion bag and engaging the anti-disengagement mechanism, (2) adjusting the pressure as required, (3) pressing the inflation button to initiate infusion, and (4) allowing automatic shutoff upon completion. The device is portable and can be secured with Velcro straps to the patient’s arm or various surfaces, including the ground or a stretcher (Figures 1C,D). It maintains stable infusion performance even under turbulent conditions, making it suitable for use in complex disaster-site environments. Detailed operation videos are provided in Supplementary Video 1.

Ten male Bama miniature pigs (specific-pathogen-free [SPF]; age: 12–18 months; body weight: 25–31 kg; purchased from Changzhou Beiwang Biotechnology Co., Ltd., Changzhou, China) were used in this study. The animals were grouped according to body weight and housed in individual cages with ad libitum access to food and water. They were fasted for 12 h before the experiment but allowed ad libitum access to water. The animals were randomly assigned to two groups (*n* = 5 per group) using a random number table.

**Figure 2:**
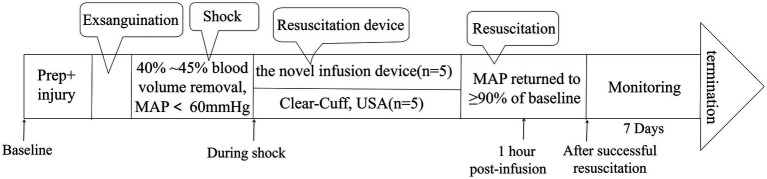
Timeline of experimental procedures.

The sample size was estimated *a priori* using PASS 11.0 statistical software. Based on our preliminary experiment results, the primary outcome measure was defined as the difference in infusion rate between the two groups. With a significance level (*α*) set at 0.05 and a statistical power (1–*β*) of 0.80, the calculation indicated that a minimum sample size of 5 animals per group was required to detect a clinically meaningful difference in infusion rate (effect size = 0.8). The randomization sequence was generated by an independent statistician who was not involved in animal grouping, data collection, or outcome assessment. Animals were numbered sequentially according to their body weight, and the random number table was used to assign each animal to either the experimental group or the control group. Group allocation was concealed using sealed, opaque envelopes, which were opened only after the successful establishment of the hemorrhagic shock model to avoid selection bias. The experimental timeline is shown in Figure 2.

The experimental group received fluid resuscitation using the portable sealed positive pressure infusion device described above. The control group received fluid resuscitation using a Clear-Cuff pressure infuser bag (model MX4705; Smiths Medical ASD, Inc., Minneapolis, MN, United States). The resuscitation solution consisted of 500 mL of 6% hydroxyethyl starch 130/0.4 in sodium chloride injection (National Medical Products Administration approval number H20103246; Beijing Fresenius Kabi Pharmaceutical Co., Ltd., Beijing, China). This colloidal solution is commonly used as a resuscitation fluid in porcine hemorrhagic shock models. Previous studies have shown that it effectively improves circulatory status during resuscitation in the Bama miniature pig hemorrhagic shock model (19, 20). We specifically selected HES for this study because it remains one of the colloids still carried by many prehospital systems in China and other regions, and its higher viscosity allowed us to evaluate device performance under challenging real-world conditions that are relevant to the intended use scenario. However, this choice was solely to assess the device’s infusion capabilities and does not imply endorsement of HES for clinical practice, as current international guidelines strongly discourage its use due to associations with renal injury, coagulopathy, and increased mortality in critically ill patients (21, 22). The study’s primary focus is on the device’s engineering and feasibility, independent of the fluid type.

### 2.5 Construction of the hemorrhagic shock model

On the day of the experiment, anesthesia was induced via intramuscular injection into the gluteus maximus muscle of tiletamine-zolazepam (Zoletil 50; 50 mg/vial; Virbac, Carros, France) at a dose of 5 mg/kg. Anesthesia was maintained with intermittent intravenous boluses of tiletamine-zolazepam at 0.5 mg/kg via the auricular vein. Subsequent procedures were initiated once the animals exhibited diminished corneal reflexes and muscle relaxation.

**Figure 3:**
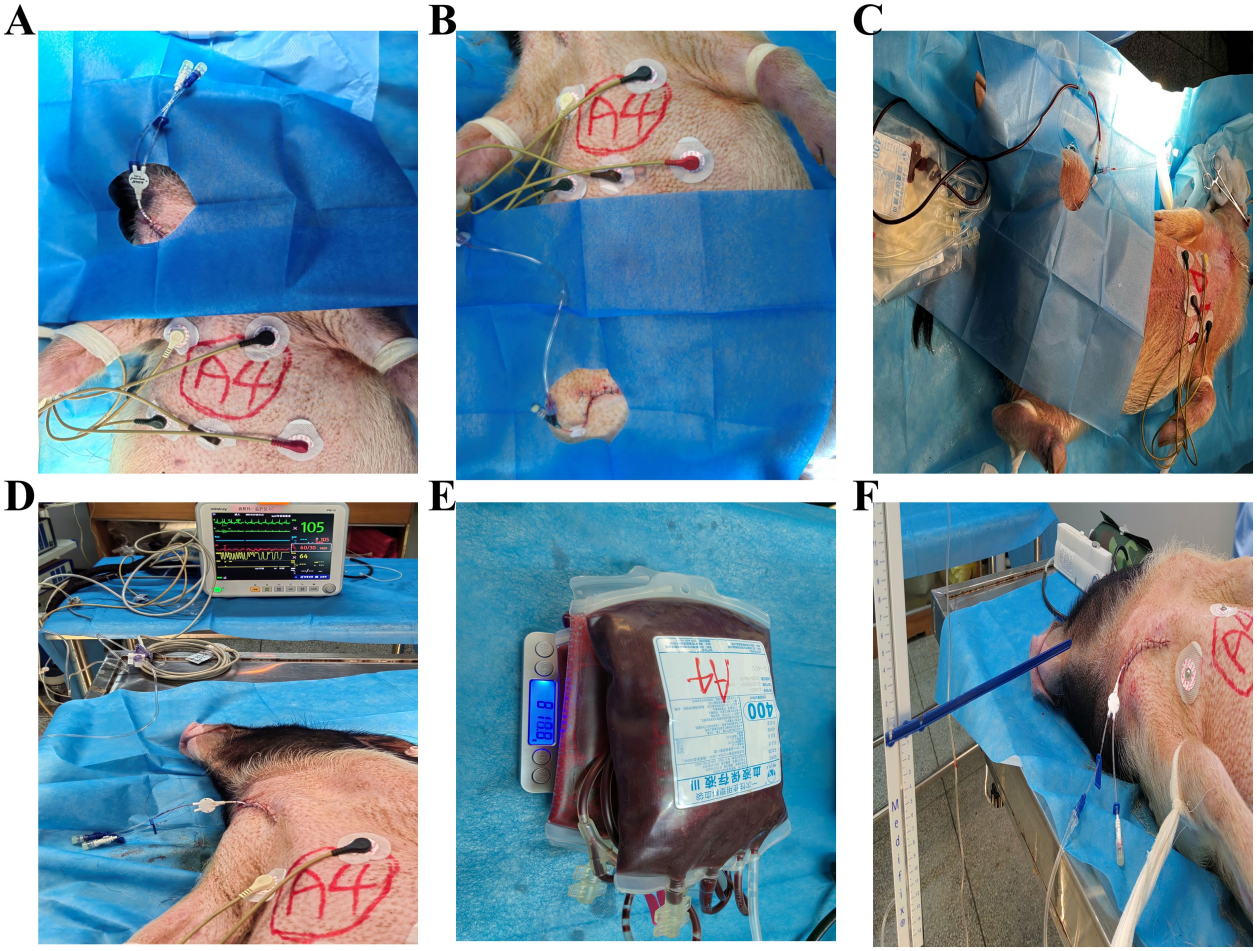
Establishment of the hemorrhagic shock model in Bama miniature pigs. **(A)** Central venous cannulation via the external jugular vein. **(B)** Femoral artery cannulation for blood withdrawal and pressure monitoring. **(C)** Induction of hemorrhagic shock via controlled blood loss. **(D)** Real-time monitoring of vital signs during shock phase. **(E)** Measurement of total blood loss by weighing collection bags. **(F)** Assessment of central venous pressure using a precision pressure gauge.

After stable anesthesia was achieved, the animal’s neck was disinfected and draped. A right internal jugular vein catheterization was then performed using the Seldinger technique with a 7 Fr double-lumen central venous catheter (Arrow; Teleflex, Wayne, PA, United States; Figures 3A,F). After the catheter tip was positioned at the inlet of the right atrium, two connections were established using a three-way connector: one for fluid administration and the other for monitoring central venous pressure with a precision digital pressure gauge (accuracy, ±0.5 mmHg).

After the internal jugular vein catheterization was completed, the right inguinal region of the animals was disinfected and shaved. Subsequently, a 2-cm skin incision was made, the femoral artery was dissected free, and a 20 G × 25 mm indwelling needle (Jierui; Weihai Jierui Medical Products Co., Ltd., Weihai, Shandong, China) was inserted (Figure 3B). The arterial catheter was connected to an arterial pressure sensor (MMBPT SA20) to enable real-time monitoring of arterial blood pressure, heart rate, and oxygen saturation (Figure 3D).

**Figure 4:**
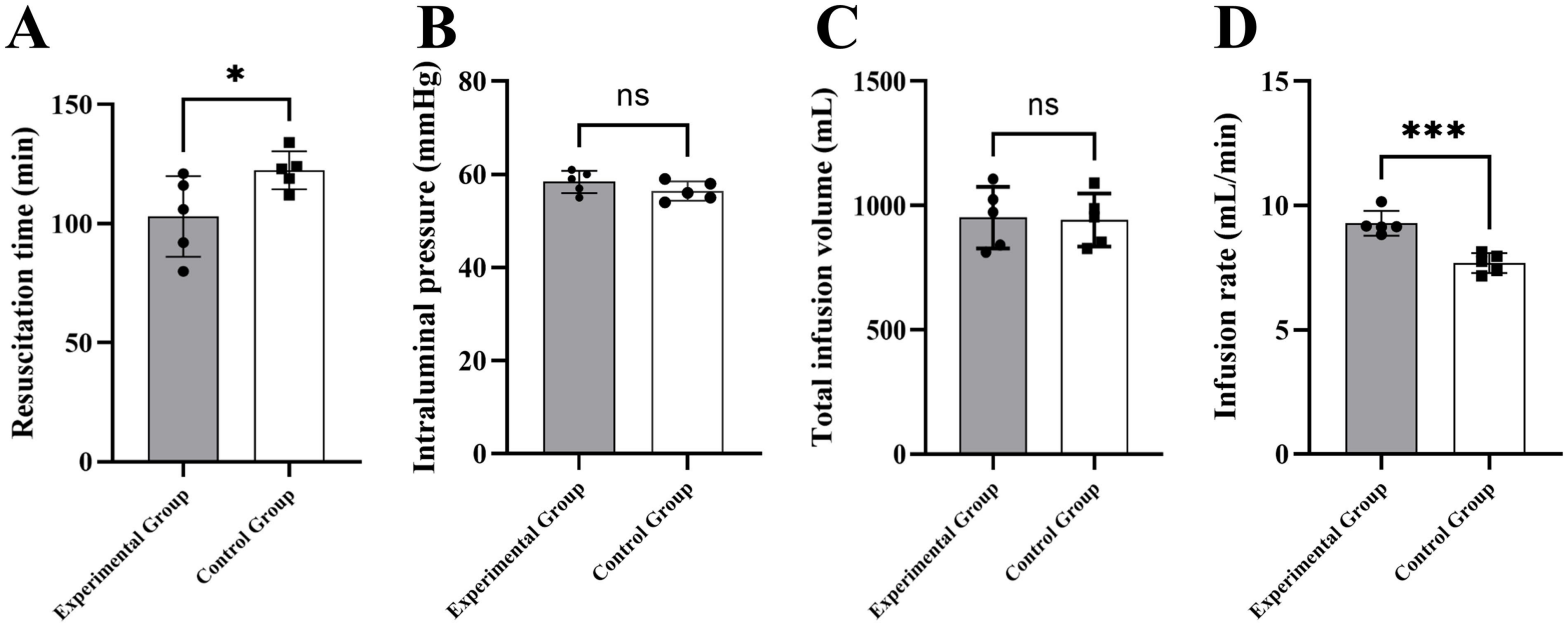
Comparison of resuscitation efficiency and infusion safety between the experimental and control groups. **(A)** Resuscitation time. **(B)** Peak intraluminal pressure. **(C)** Total infusion volume. **(D)** Average infusion rate.

A hemorrhagic shock model was established as previously described (21). Blood was withdrawn via the femoral artery catheter at a controlled rate of 2.5–3.0 mL/kg/min until approximately 40–45% of estimated total blood volume (70 mL/kg body weight) had been removed, resulting in a sustained MAP <60 mmHg in all animals. The collected blood was stored in a blood collection bag for potential reinfusion. Following the initial hemorrhage phase, small amounts of blood were reinfused or further withdrawn as needed to maintain MAP <60 mmHg for 30 min. The model was considered successful when MAP remained <60 mmHg for at least 30 min (Figures 3C,E). All procedures were performed under strict aseptic conditions. After catheter placement, the incision site was covered with a sterile dressing to minimize the risk of infection. The animals were randomly assigned to two groups: the experimental group received fluid resuscitation using the portable sealed positive pressure infusion device described above, while the control group received fluid resuscitation using the Clear-Cuff pressure infuser bag (model MX4705; Smiths Medical ASD, Inc., Minneapolis, MN, United States). The animals were selected from their housing cages by researchers blinded to group allocation. For each experiment, two sets of resuscitation infusion devices were prepared in advance. Group assignments were revealed by the principal investigator only after successful establishment of the hemorrhagic shock model, at which point the corresponding infusion device was selected for fluid resuscitation (Figure 4).

### 2.6 Fluid resuscitation protocol

In the experimental group, fluid resuscitation was performed using the portable sealed positive pressure infusion device described above. First, 500 mL of 6% hydroxyethyl starch 130/0.4 in sodium chloride injection was placed into the pressure chamber. Second, the pressure-reducing valve was adjusted to 300 mmHg. Third, the infusion was initiated by pressing the inflation button, with an initial flow rate of 10 mL/min.

The control group received fluid resuscitation using the Clear-Cuff pressure infuser bag. First, the pressure bag was manually squeezed until the pressure gauge indicated 300 mmHg. Second, the flow regulator was adjusted to a flow rate of 10 mL/min. Except for the different infusion devices, all other procedures were performed by the same medical team following standardized protocols. Detailed experimental procedures and illustrations are provided in Supplementary Figure 1.

The standardized procedure was as follows: First, prior to infusion, the deep venous catheter access site and the three-way connector were each wiped three times with 75% ethanol to ensure aseptic conditions. Next, the 6% hydroxyethyl starch 130/0.4 in sodium chloride injection was visually inspected to confirm the absence of precipitation or flocculent matter and to verify that the solution was within its expiration date. Then, the gravity-venting bottle was inverted, and the flow regulator was fully opened to purge air from the infusion line. Finally, the pressure was adjusted to 300 mmHg, the initial infusion rate was set to 10 mL/min, and the infusion was initiated. During the infusion, the pressure within the infusion line was recorded in real time using a precision digital pressure gauge. Successful resuscitation was defined as the restoration of MAP to ≥90% of the pre-shock baseline value, maintained for at least 15 min (23). The baseline value was defined as the mean MAP measured over the 30 min preceding establishment of the hemorrhagic shock model and independently verified by two critical care physicians.

### 2.7 Monitoring parameters

Vital signs and key blood parameters were monitored at four predefined time points: baseline, during shock, 1 h after fluid resuscitation, and upon successful resuscitation. Vital signs, including respiratory rate, blood pressure, heart rate, blood oxygen saturation, and central venous pressure (CVP), were continuously monitored using an electrocardiogram monitor and arterial pressure sensors. Key blood parameters, including prothrombin time (PT), activated partial thromboplastin time (APTT), hemoglobin (Hb), platelet count (PLT), and serum creatinine (SCr), were assessed from peripheral venous blood samples (1 mL) collected from the auricular vein. Blood samples were collected into standard blood collection tubes, stored at 4 °C, and analyzed within 60 min of collection.

The infusion efficiency of the two devices was evaluated by comparing the time required to achieve successful resuscitation and the total volume of fluid administered. The intraluminal pressure exerted by the infusion fluid on the walls of the deep venous catheter was monitored throughout the infusion process using a precision digital pressure gauge to assess the safety of the different infusion devices. After fluid resuscitation, once MAP had stabilized and vital signs were restored, definitive surgery was performed to evaluate wound status and repair injured tissues.

A 7-day postoperative follow-up was conducted to monitor survival and clinical status. Predefined humane endpoints included inability to eat for ≥24 h, persistent recumbency, body weight loss >20%, or unrelieved pain despite analgesia (buprenorphine at 0.01 mg/kg intramuscularly every 12 h, indicated for signs of pain such as vocalization, reluctance to move, or abnormal posture). Animals reaching any humane endpoint, or surviving to the end of the 7-day observation period, were humanely euthanized by intravenous administration of sodium pentobarbital (150 mg/kg body weight) via the indwelling central venous catheter. Death was confirmed by the absence of corneal reflex, heartbeat, and respiration for at least 5 min. All deaths—whether spontaneous or by euthanasia—were independently evaluated by two blinded veterinarians to determine causality related to the infusion device. The study was conducted in strict accordance with the Regulations on the Administration of Laboratory Animals of the People’s Republic of China and institutional animal welfare protocols.

### 2.8 Statistical analysis

All data were organized using Microsoft Excel, statistically analyzed with R software (version 4.4.1; The R Foundation for Statistical Computing, Vienna, Austria), and visualized using GraphPad Prism (version 9.5; GraphPad Software, Boston, MA, United States). Continuous variables were presented as mean ± standard deviation (SD) with exact 95% confidence intervals (95% CIs) reported for all primary outcomes. For repeatedly measured outcomes (including respiratory rate, mean arterial pressure, heart rate, central venous pressure, activated partial thromboplastin time, hemoglobin, platelet count, and serum creatinine) assessed at four time points (baseline, shock phase, 1 h post-resuscitation, and upon completion of resuscitation), a two-way repeated-measures analysis of variance (ANOVA) was applied using the aov function in R’s stats package. This model incorporated two fixed effects: group (experimental vs. control) and time point, as well as the group-time interaction (to examine whether the effect of time differed between groups). Individual animals were treated as the within-subject factor (to account for repeated measurements on the same animal). For between-group comparisons of non-repeated outcomes (resuscitation time, total infusion volume, intraluminal pressure, and infusion rate), independent samples t-tests were used (these outcomes were measured once per animal and thus did not involve repeated measurements). A Bonferroni correction was performed for all post-hoc pairwise comparisons (both within-group time-point comparisons and between-group time-matched comparisons) to address the issue of multiple comparisons. Survival during the 7-day follow-up period was analyzed using the Kaplan–Meier method, with differences between groups compared using the log-rank test. A *p* value <0.05 was considered statistically significant.

## 3 Results

### 3.1 Baseline characteristics and hemodynamic and hematologic responses

Ten healthy adult male Bama miniature pigs were enrolled and randomly assigned to the experimental or control group (*n* = 5/group). Mean body weights were 25.88 ± 2.46 kg in the experimental group and 26.28 ± 0.55 kg in the control group, with no significant differences between groups (*p* = 0.341), indicating comparable baseline characteristics (Table 1). All animals successfully established the hemorrhagic shock model and survived until experiment completion (100% immediate survival post-resuscitation). Hemorrhage volumes to achieve successful model establishment were comparable between groups: experimental group 778.2 ± 30.76 [95% CI: 740.0, 816.4] mL, control group 764.5 ± 26.59 [95% CI: 731.5, 797.5] mL, with no significant differences between groups (*p* > 0.05). At 1 week post-experiment, survival rates were 80% in both groups (one death and four survivors per group), with no significant difference (*p* = 0.937, see Supplementary Figure 2 for Kaplan–Meier survival curves).

**Table 1:** Comparison of vital signs and laboratory indices between the two groups at different time periods.

Parameters	Groups	Baseline	Shock phase	*p* (vs. Baseline)	1 hour post-infusion	*p* (vs. Baseline)	Resuscitation	*p* (vs. Baseline)
RR (breaths/min)	Experimental group	18.8 ± 1.92 (16.41, 21.19)	34.4 ± 8.17* (24.25, 44.55)	0.0008	24.6 ± 3.44 (20.33, 28.87)	>0.9999	23.8 ± 4.38 (18.36, 29.24)	>0.9999
	Control group	20.2 ± 3.90 (15.36, 25.04)	34.8 ± 7.05* (26.05, 43.55)	0.0018	22.2 ± 6.30 (14.38, 30.02)	>0.9999	21.2 ± 4.87 (15.16, 27.4)	>0.9999
	*p* (Group Comparison)	>0.9999	>0.9999		>0.9999		>0.9999	
MAP (mmHg)	Experimental group	118.84 ± 18.74 (95.57, 142.11)	47.88 ± 9.45* (36.14, 59.62)	<0.0001	95.29 ± 11.38 (81.17, 109.42)	0.0322	102.26 ± 9.19 (90.84, 113.68)	0.2600
	Control group	111.14 ± 16.78 (90.30, 131.98)	49.26 ± 6.47* (41.23, 57.29)	<0.0001	96.08 ± 12.84 (80.14, 112.02)	0.3900	99.04 ± 9.96 (86.68, 111.40)	0.8071
	*p* (Group Comparison)	>0.9999	>0.9999		>0.9999		>0.9999	
Heart rate (beats/min)	Experimental group	114.2 ± 6.38 (106.28, 122.12)	144.8 ± 7.05* (136.05, 153.55)	<0.0001	119.8 ± 7.16 (110.92, 128.68)	>0.9999	116 ± 6.04 (108.50, 123.50)	>0.9999
	Control group	115.6 ± 8.96 (104.47, 126.73)	142.2 ± 6.42* (134.23, 150.17)	<0.0001	118.8 ± 4.66 (113.02, 124.58)	>0.9999	120.2 ± 11.52 (105.90, 134.50)	>0.9999
	*p* (Group Comparison)	>0.9999	>0.9999		>0.9999		>0.9999	
SPO2(%)	Experimental group	98.4 ± 1.14 (96.98, 99.82)	86.4 ± 2.97* (82.72, 90.08)	<0.0001	95.6 ± 1.82 (93.34, 97.86)	0.4897	96.0 ± 2.12 (93.37, 98.63)	0.7986
	Control group	98.8 ± 0.45 (98.24, 99.36)	86.4 ± 3.78* (81.70, 91.10)	<0.0001	96.4 ± 3.05 (92.61, 100.19)	0.7986	96.6 ± 2.61 (93.36, 99.84)	>0.9999
	*p* (Group Comparison)	>0.9999	>0.9999		>0.9999		>0.9999	
CVP (mmHg)	Experimental group	9.6 ± 2.61 (6.36, 12.84)	2.2 ± 1.92* (−1.88, 4.59)	<0.0001	7 ± 1.58 (5.04, 8.96)	0.2457	7.4 ± 1.67 (5.32, 9.48)	0.4855
	Control group	9.6 ± 1.52 (7.72, 11.48)	2.2 ± 2.39* (−0.76, 5.16)	<0.0001	7.2 ± 1.30 (5.58, 8.82)	0.3480	8.4 ± 2.07 (5.83, 10.97)	>0.9999
	*p* (Group Comparison)	>0.9999	>0.9999		>0.9999		>0.9999	
Blood PT (sec)	Experimental group	11.3 ± 0.70 (10.44, 12.16)	11.42 ± 0.78 (10.45, 12.39)	>0.9999	11.14 ± 0.93 (9.99, 12.29)	>0.9999	11.12 ± 1.09 (9.76, 12.48)	>0.9999
	Control group	11.34 ± 0.92 (10.19, 12.49)	11.94 ± 0.63 (11.15, 12.73)	>0.9999	11.48 ± 1.21 (9.98, 12.98)	>0.9999	11.36 ± 0.76 (10.42, 12.30)	>0.9999
	*p* (Group Comparison)	>0.9999	>0.9999		>0.9999		>0.9999	
Blood aPTT (sec)	Experimental group	29.32 ± 5.25 (22.80, 33.84)	26.16 ± 5.04 (19.90, 32.4)	>0.9999	20.5 ± 4.00* (15.54, 25.46)	0.0282	20.16 ± 4.40* (14.70, 25.62)	0.0209
	Control group	30.88 ± 4.56 (25.22, 36.54)	29.44 ± 5.14 (23.06, 35.82)	>0.9999	22.48 ± 3.90* (17.64, 27.32)	0.0408	22.46 ± 4.22* (17.22, 27.70)	0.0401
	*p* (Group Comparison)	>0.9999	>0.9999		>0.9999		>0.9999	
Hb (g/L)	Experimental group	109.6 ± 5.5 (102.77, 116.43)	109.6 ± 2.88 (106.02, 113.18)	>0.9999	99.2 ± 9.39 (87.54, 110.86)	0.0657	100.6 ± 6.62 (92.38, 108.82)	0.1250
	Control group	111.8 ± 7.05 (103.05, 120.55)	107.2 ± 3.83 (102.44, 111.96)	>0.9999	98.1 ± 3.67 (87.14, 109.45)	0.0657	94.4 ± 4.51* (88.81, 99.99)	0.0003
	*p* (Group Comparison)	>0.9999	>0.9999		>0.9999		0.4149	
PLT (×10^9^/L)	Experimental Group	460.6 ± 13.85 (443.40, 477.80)	425.4 ± 25.59 (393.63, 457.17)	0.3702	422.4 ± 35.70 (378.08, 466.72)	0.2614	421.6 ± 34.29 (379.02, 464.18)	0.2377
	Control group	461.2 ± 26.11 (428.78, 493.62)	417.4 ± 26.43 (384.59, 450.21)	0.1314	412.6 ± 32.99 (371.64, 453.56)	0.0703	407 ± 29.05* (370.93, 443.07)	0.0327
	*p* (Group Comparison)	>0.9999	>0.9999		>0.9999		>0.9999	
SCr (μmol/L)	Experimental Group	71.34 ± 9.51 (59.53, 83.15)	67.48 ± 7.46 (58.21, 76.75)	>0.9999	67.46 ± 10.04 (54.99, 79.93)	>0.9999	67.22 ± 9.95 (54.87, 79.57)	>0.9999
	Control Group	72.28 ± 10.88 (58.77, 85.79)	68.24 ± 12.31 (52.95, 83.53)	>0.9999	66.06 ± 10.63 (52.86, 79.26)	>0.9999	66.56 ± 10.5 (53.52, 79.60)	>0.9999
	*p* (Group Comparison)	>0.9999	>0.9999		>0.9999		>0.9999	

Experimental group: the portable sealed positive pressure infusion device. Control group: Clear-Cuff pressure infuser bag. RR, Respiratory Rate; MAP, Mean Arterial Pressure; SPO2, Peripheral Oxygen Saturation; CVP, Central Venous Pressure; PT, Prothrombin Time; aPTT, Activated Partial Thromboplastin Time; Hb, Hemoglobin; PLT, Platelets; SCr, Serum Creatinine. Data are expressed as means ± SD with 95% confidence intervals (95% CIs). ^*^p < 0.05 compared to corresponding baseline (mixed-effects model with Bonferroni post-hoc correction); ^#^*p* < 0.05 between experimental and control groups at the same time point (mixed-effects model with Bonferroni *post-hoc* correction).

At baseline, both groups showed comparable values for all measured parameters, with no significant differences between groups (*p* > 0.05; Table 1). After hemorrhagic shock model establishment, animals in both groups showed characteristic changes, including increases in respiratory rate and heart rate (*p* < 0.05) and decreases in CVP and MAP compared with baseline (*p* < 0.05). The hemorrhagic shock model was successfully established in both groups, with comparable severity. After resuscitation, animals in both groups showed increases in MAP and CVP compared with shock levels (*p* < 0.05). MAP recovered to >90% of baseline and remained stable for >15 min, meeting resuscitation success criteria. No significant differences were observed between groups in these parameters (*p* > 0.05; Table 1). The temporal changes in these vital signs (HR, SpO₂, MAP, RR) at each time point are shown in Supplementary Figure 3.

PT remained stable in both groups at baseline, during shock, 1 h post-resuscitation, and after successful resuscitation (*p* > 0.05). APTT shortened in both groups during shock compared with baseline (*p* < 0.05) and further shortened during resuscitation (*p* < 0.05). Hb levels remained stable in both groups during shock but decreased 1 h post-resuscitation and after successful resuscitation. The control group showed a greater Hb reduction after successful resuscitation than the experimental group. PLT decreased in both groups during shock, with no further changes after resuscitation (*p* > 0.05). SCr levels decreased during shock (*p* < 0.05) and stabilized after resuscitation (*p* > 0.05), with no differences between groups at corresponding time points (*p* > 0.05; Table 1). The detailed temporal changes in CVP, Hb, PT, APTT, PLT, and SCr are illustrated in Supplementary Figure 4 and Supplementary Figure 5.

### 3.2 Infusion efficiency comparison between devices

After resuscitation, the experimental group required less time to achieve success (103.0 ± 16.97 [95% CI: 81.93, 124.07] minutes) than the control group (122.4 ± 8.02 min [95% CI: 112.44, 132.36]; *p* = 0.0496). No difference was observed in total resuscitation fluid volume between groups (experimental: 951.4 ± 124.07 mL; control: 942.4 ± 106.67 mL; *p* > 0.05). The experimental group’s infusion rate (9.29 ± 0.50 mL/min) was higher than the control group’s (7.68 ± 0.40 mL/min; *p* < 0.05; Table 2).

**Table 2:** Comparison of infusion efficiency and device safety metrics.

Group	Resuscitation time (min)	Total infusion volume (mL)	Intraluminal pressure (mmHg)	Infusion rate (mL/min)
Experimental Group	103.0 ± 16.97 (81.93, 124.07)	951.4 ± 124.07 (797.35, 1105.44)	58.4 ± 2.41 (55.41, 61.39)	9.29 ± 0.50 (8.67, 9.91)
Control Group	122.4 ± 8.02 (112.44, 132.36)	942.4 ± 106.67 (809.5, 1074.85)	56.4 ± 2.07 (53.83, 58.97)	7.68 ± 0.40 (7.18, 8.18)
t value	−2.311	0.123	1.407	5.588
*p* value	0.0496	0.905	0.197	<0.001

Experimental group: the portable sealed positive pressure infusion device. Control group: Clear-Cuff pressure infuser bag. Data are expressed as mean ± SD, 95% CI.

### 3.3 Safety evaluation of infusion devices

During infusion, intraluminal pressure monitoring showed mean peak pressures of 58.4 ± 2.41 mmHg in the experimental group and 56.4 ± 2.07 mmHg in the control group, with no difference between groups (*p* > 0.05). No pressure-related complications, such as fluid extravasation or hematoma at the puncture site, occurred in either group.

During the 7-day postoperative follow-up, one animal died in each group. No evidence of air or fat embolism was observed during follow-up. Catheters remained patent, puncture sites healed appropriately, and no complications such as hemolysis or major hemorrhage were detected. Vital sign changes preceding deaths were consistent with hemorrhagic shock progression; blinded veterinarians attributed both deaths to model-related delayed physiological decompensation following severe hemorrhage and resuscitation rather than to the experimental intervention.

## 4 Discussion

This study addressed challenges in intravenous fluid administration for prehospital care in complex environments, such as war zones and natural disasters, by developing a portable sealed positive-pressure infusion device. During operation, the pressure generator provides continuous, controllable fluid propulsion for stable infusion under low-perfusion conditions; the pressure chamber maintains uniform pressure transmission in the sealed environment, preventing vascular damage or device leakage from sudden pressure changes; the infusion system is compatible with standard tubing, and the integrated gravity-venting drip chamber enables automatic air purging, reducing risks and air embolism threats in prehospital care. To validate efficacy, this study used a hemorrhagic shock model in Bama miniature pigs for comparative analysis. Vital signs and blood parameters were compared between groups at baseline, shock, 1 h post-resuscitation, and successful resuscitation to evaluate device efficacy and safety. Assessments included total resuscitation time, fluid volume administered, and intraluminal pressure during infusion. Long-term outcomes were validated via 7-day postoperative survival rates, providing evidence for further translational research.

This study found no statistically significant differences in vital signs, key blood parameters, or 7-day survival rates between the portable sealed positive-pressure infusion device and the traditional pressure infuser bag during fluid resuscitation in a Bama miniature pig model of hemorrhagic shock (*p* > 0.05). These results confirm that the novel device’s basic resuscitation efficacy is comparable to that of traditional equipment. However, under identical infusion pressure conditions, the portable sealed positive-pressure infusion device, via stable and controllable positive-pressure propulsion, significantly shortened total resuscitation time, increased infusion rate, and maintained stable intraluminal pressure compared with the traditional pressure infuser bag (*p* < 0.05). These findings indicate that the device replenishes circulating blood volume more rapidly, which may be advantageous in time-critical prehospital resuscitation.

Safety assessments revealed no statistically significant difference in mean peak intraluminal pressure between groups during infusion (*p* > 0.05). Further analysis of blood parameters indicated that Hb levels decreased more significantly in the control group upon successful resuscitation compared with the experimental group (*p* < 0.05), likely due to longer resuscitation time and more pronounced hemodilution in the control group. PLT decreased slightly in both groups during shock, with no further significant changes after fluid resuscitation (*p* > 0.05). SCr levels decreased slightly during the shock phase (*p* < 0.05) and returned to near-baseline levels post-resuscitation in both groups, with no significant intergroup differences at any time point. However, SCr is a relatively insensitive and late marker of acute kidney injury. Therefore, the present data do not allow conclusions regarding potential subtle renal injury or differences in early renal perfusion between devices.

Regarding environmental adaptability, the device requires no electrical power, features a compact design, offers simple operation, maintains continuous infusion during patient transport, and facilitates easy fluid exchange. These attributes make it potentially suitable for resource-limited scenarios, such as disasters and wars, where traditional gravity-fed infusion sets are limited by patient positioning and electric pressure devices depend on power sources, often leading to missed resuscitation opportunities due to operational delays (8). Furthermore, its automatic pressurization and alarm-triggered flow cutoff functions minimize reliance on specialized personnel, thereby reducing manpower needs and facilitating prehospital emergency care requirements for rapid response and simplified operation.

Although current resuscitation strategies for hemorrhagic shock have shifted from simple fluid resuscitation to prioritizing coagulation function protection, multiple studies confirm that large-volume crystalloid infusion induces complications such as intra-abdominal hypertension, multiple organ failure, and dilutional coagulopathy, whereas colloid infusion causes renal impairment and coagulation factor interference (21, 22, 24–27). Balanced blood component transfusion (1:1:1 or 1:1:2) and whole blood transfusion remain mainstream recommendations for hemorrhagic shock resuscitation, as they address hypovolemia and coagulation disorders simultaneously (28). This study observed that rapid resuscitation with hydroxyethyl starch solution was associated with further APTT shortening. These descriptive findings generate hypotheses regarding potential colloid effects on coagulation parameters but must be interpreted cautiously, as APTT alone is insufficient to infer overall coagulation status or imply clinical benefit or harm without viscoelastic testing (e.g., TEG or ROTEM). However, the device’s technical advantages—rapidity, portability, and safety—are independent of the resuscitation fluid, and it can serve as providing an efficient infusion platform for any fluid type. Importantly, while the device improved infusion stability and rate, its adjustable pressure settings make it particularly suitable for permissive hypotensive resuscitation strategies, which aim to maintain a lower MAP (e.g., 65–70 mmHg) to minimize re-bleeding risks in uncontrolled hemorrhage (3). By providing consistent, controlled flow without excessive rapidity, the device helps avoid the adverse effects of aggressive volume resuscitation, such as disruption of hemostatic clots, dilutional coagulopathy, and hypothermia (16, 17). This aligns with current guidelines recommending delayed aggressive resuscitation in prehospital settings to improve outcomes in trauma patients (22). In our model, the stable pressure prevented fluctuations that could exacerbate these issues, supporting its utility in austere environments where over-resuscitation is a common pitfall.

Therefore, future studies could evaluate the device’s efficacy in balanced blood component and whole blood transfusions to validate its pressure adaptability during blood product infusion and assess its value in sequential resuscitation strategies involving initial colloid maintenance followed by blood product correction of coagulation disorders. Such validation would be particularly relevant in prehospital settings without blood products, where rapid volume resuscitation maintains vital signs and allows time for transport to equipped facilities.

There are some limitations in the present study. First, the small sample size (n = 5 per group) may limit statistical power and the generalizability of findings, particularly for detecting subtle effects, though it was justified by pilot data and power calculations; larger cohorts would enhance robustness in future validations. Second, we used hydroxyethyl starch (HES 130/0.4) as the resuscitation fluid. Although HES was chosen because it is still widely carried in many prehospital emergency medical systems in China and several other countries, and it allowed us to test our device under high-viscosity conditions that represent a worst-case scenario for infusion speed, we fully acknowledge the current international consensus and high-grade evidence against the routine use of HES in critically ill and major hemorrhage patients due to increased risks of acute kidney injury, coagulopathy, and mortality (24–27). Future studies could incorporate blood products to better mimic current human resuscitation standards and mitigate these risks. Third, APTT results were interpreted cautiously as indicators of colloid effects; accordingly, the observed coagulation changes related to HES should be viewed as hypothesis-generating rather than definitive. Viscoelastic testing (e.g., TEG/ROTEM) would provide a more comprehensive coagulopathy assessment; future studies should incorporate these to address potential HES-induced changes and prioritize guideline-recommended fluids like blood products to align with contemporary resuscitation principles. Fourth, renal function was assessed only by serum creatinine, which is an insensitive and delayed marker of acute kidney injury. No histopathological examination was performed, and more sensitive early biomarkers (e.g., NGAL, KIM-1, cystatin C) or liver enzymes were not measured. Therefore, subtle or delayed organ injury cannot be excluded. Future studies should incorporate histopathological analysis and modern kidney injury biomarkers to more rigorously evaluate long-term safety. Finally, translational challenges remain, including species differences between porcine and human physiology (e.g., in coagulation and vascular responses), the controlled laboratory setting that does not fully replicate real-world environmental stressors (e.g., extreme temperatures), and the need for further testing of the device’s performance in austere environments. The 7-day follow-up is relatively short, potentially missing delayed complications. Future research should employ larger samples, blood products, advanced coagulation assays, and human simulation studies to overcome these limitations and accelerate clinical translation.

## 5 Conclusion

In this porcine model of hemorrhagic shock, the portable sealed positive-pressure infusion device achieved significantly higher infusion rates and shorter time to reach resuscitation targets compared with the conventional pressure infuser bag under identical pressure settings. Peak intraluminal pressures remained comparable between groups with no evidence of device-related complications during the experiment. The device is electricity-independent, portable, and operable in various patient positions. These characteristics suggest potential utility in resource-limited prehospital settings, although further validation with currently recommended resuscitation fluids and eventual human studies will be required before clinical application.

## Data Availability

The raw data supporting the conclusions of this article will be made available by the authors, without undue reservation.
