# Supplementary material for: Application of a portable sealed positive pressure infusion device in a porcine model of hemorrhagic shock
Source: Front Med (Lausanne). 2026 Jan 15;13:1738724. doi: 10.3389/fmed.2026.1738724 (PMC12852321; doi:10.3389/fmed.2026.1738724)
Supplement: Supplementary file 1 [file Table_1.docx]

**Supplementary Material**

**1 Supplementary video**

Detailed operation videos of the device.

**2 Supplementary Figures**


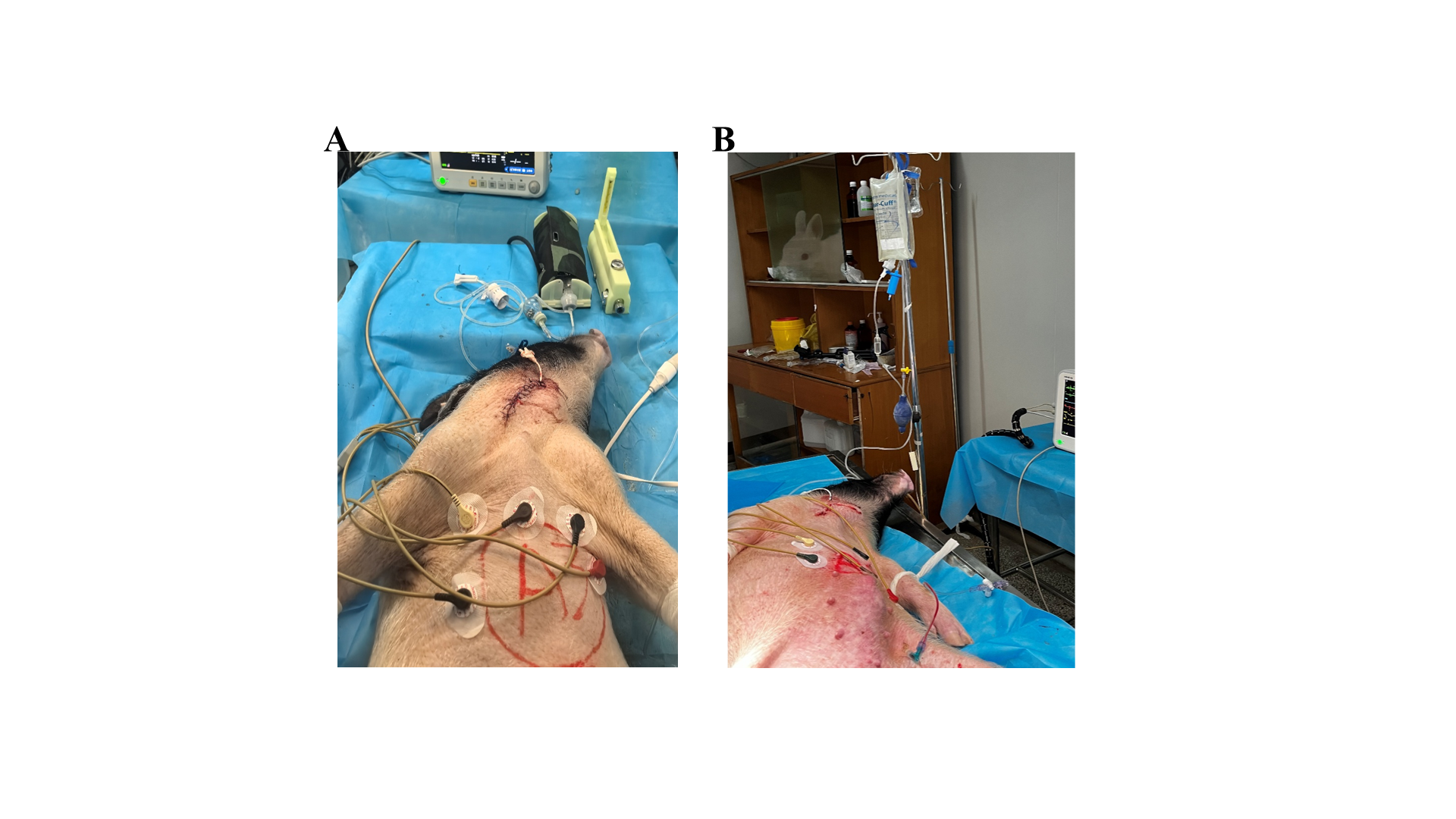


**Supplementary Figure 1** Animal resuscitation after severe hemorrhagic shock.(A) Experimental group treated with the newly developed portable closed positive-pressure infusion device.(B) Control group treated with a conventional manual pressurized infusion bag.


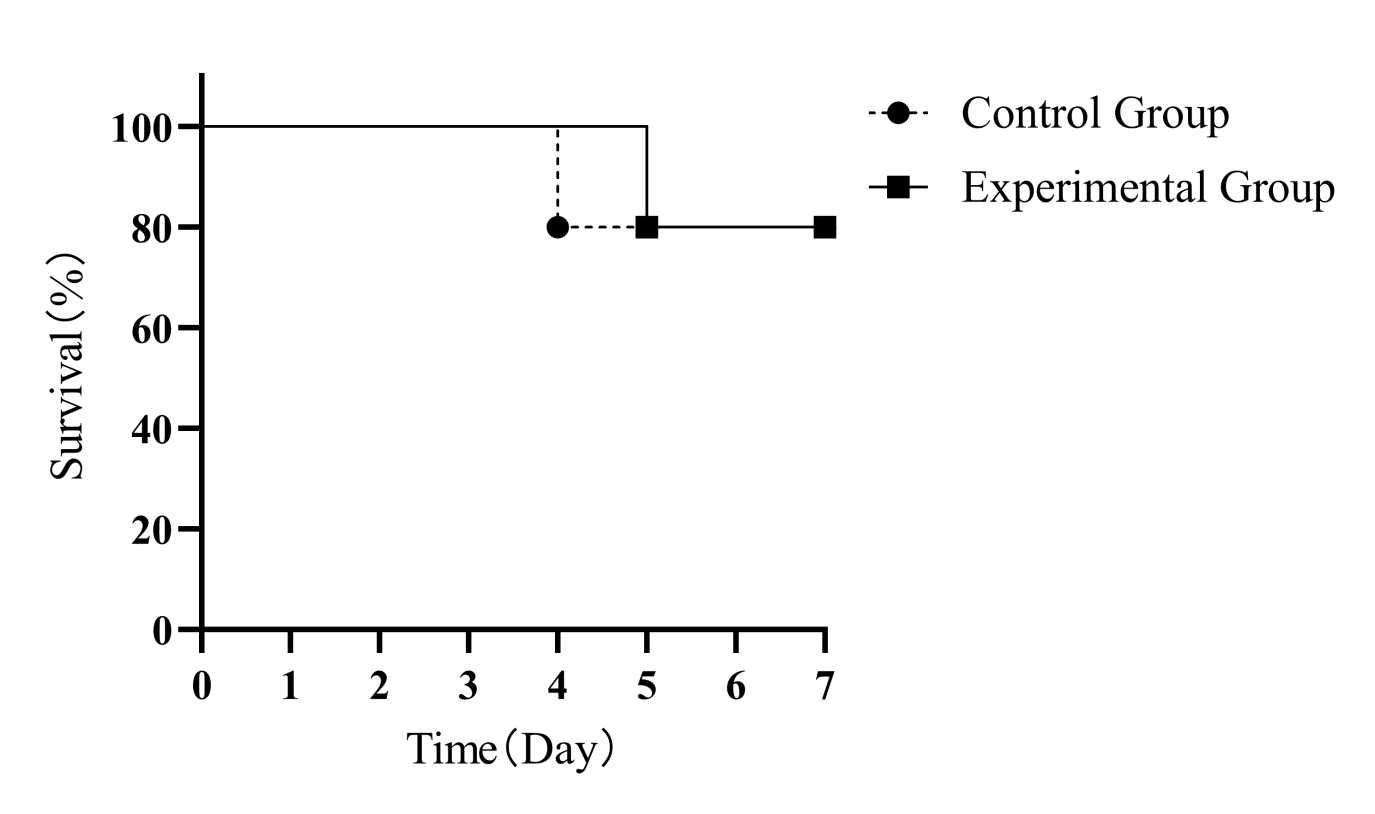


**Supplementary Figure 2** Kaplan-Meier survival curves of Bama miniature pigs with hemorrhagic shock resuscitated by the novel portable sealed positive-pressure infusion device versus conventional pressure infusion device within 7 days.


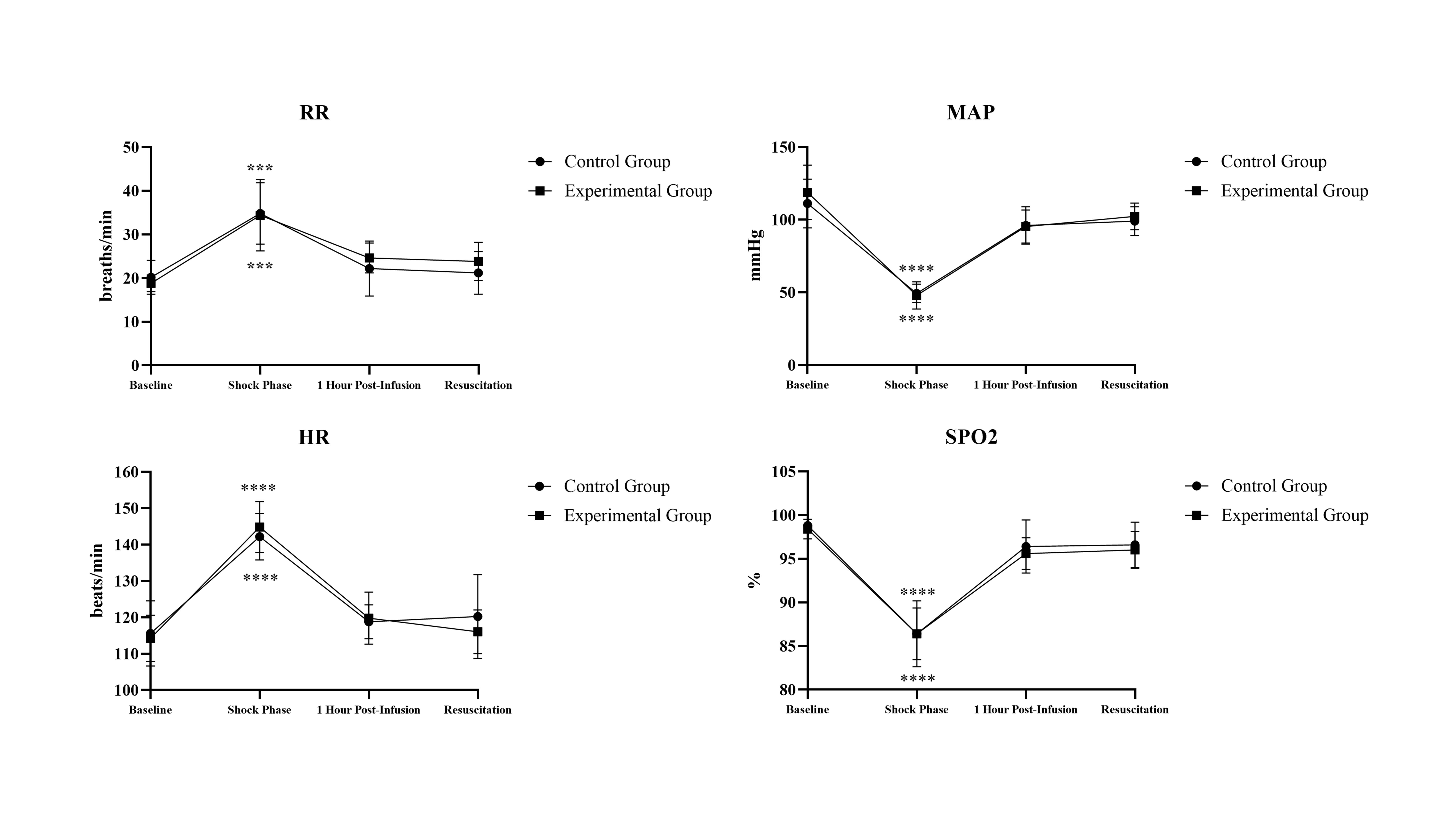


**Supplementary Figure 3** Changes of HR, SPO_2_, MAP and RR following hemorrhage and resuscitation with experimental group or control group in pigs.

Abbreviations: HR = heart rate; SPO_2_ = peripheral capillary oxygen saturation; MAP = mean arterial pressure; RR = respiratory rate.

Symbols: Filled circles (•) represent the control group; filled diamonds (♦) represent the experimental group; error bars indicate standard deviation (SD).

* = *P* < 0.05; ** = *P* < 0.01 (compared to baseline); *** = *P* < 0.001 (compared to baseline); **** = *P* < 0.0001 (compared to baseline)


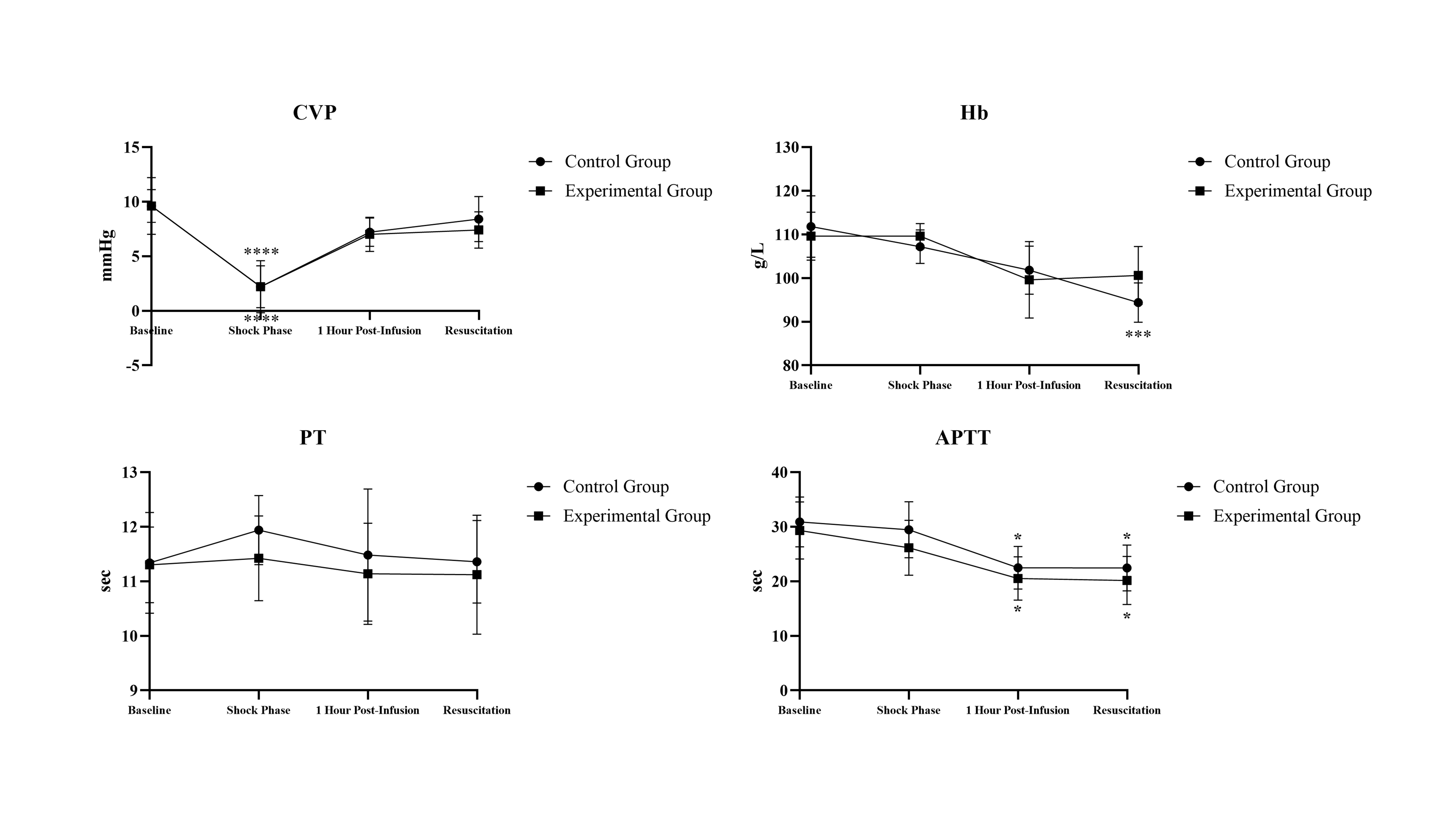


**Supplementary Figure 4** Changes of CVP, Hb, PT and APTT following hemorrhage and resuscitation with experimental group or control group in pigs.

Abbreviations: CVP = central venous pressure; Hb = hemoglobin; PT = prothrombin time; APTT = activated partial thromboplastin time.

Symbols: Filled circles (•) represent the control group; filled diamonds (♦) represent the experimental group; error bars indicate standard deviation (SD).

* = *P* < 0.05; ** = *P* < 0.01 (compared to baseline); *** = *P* < 0.001 (compared to baseline); **** = *P* < 0.0001 (compared to baseline)


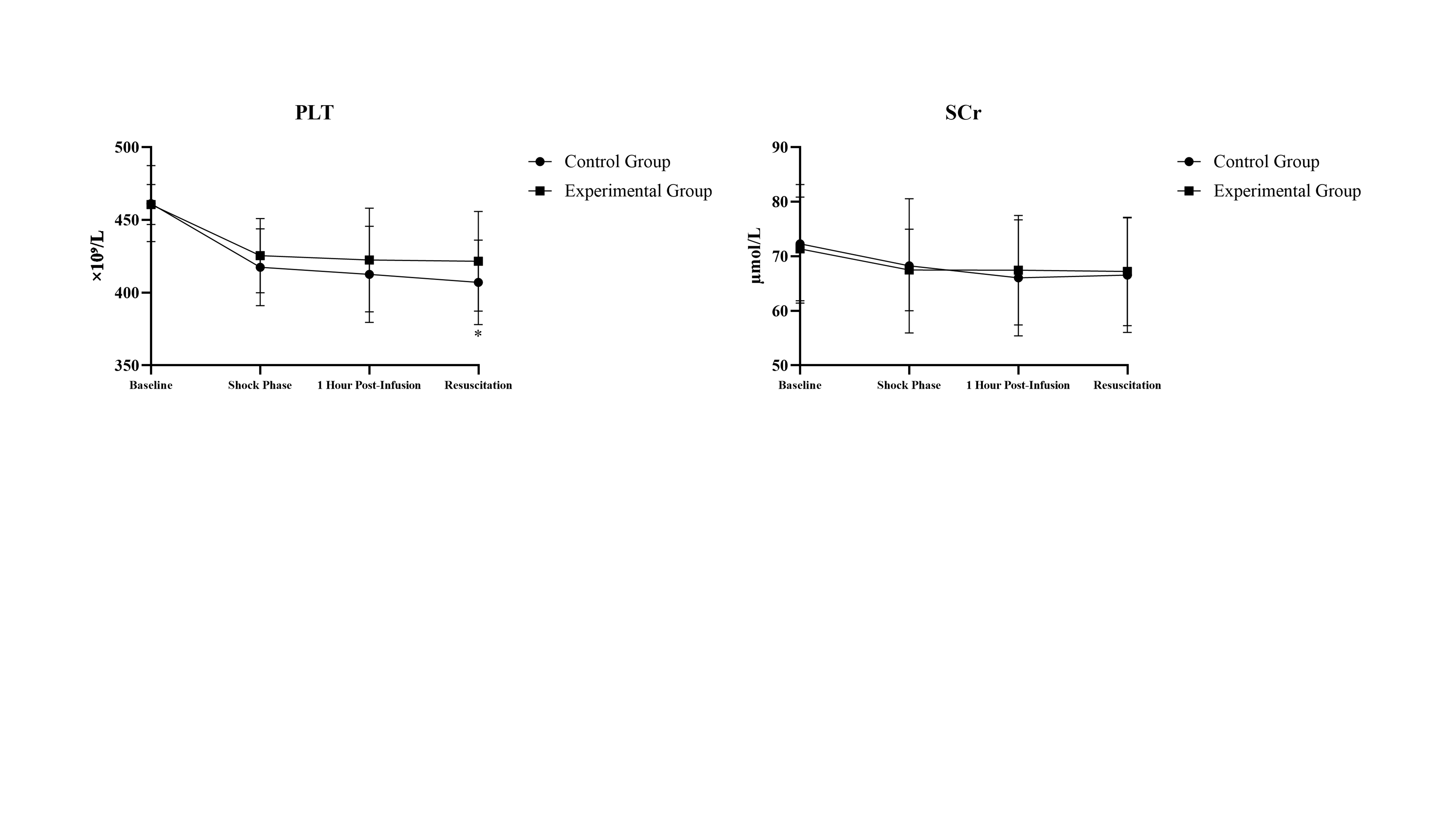


**Supplementary Figure 5** Changes of PLT and SCr following hemorrhage and resuscitation with experimental group or control group in pigs.

Abbreviations: PLT = platelet count; SCr = serum creatinine.

Symbols: Filled circles (•) represent the control group; filled diamonds (♦) represent the experimental group; error bars indicate standard deviation (SD).

* = *P* < 0.05; ** = *P* < 0.01 (compared to baseline); *** = *P* < 0.001 (compared to baseline); **** = *P* < 0.0001 (compared to baseline)

**3 Supplementary Tables**

**Supplementary Table 1** Comparison of the Homemade Portable Sealed Positive Pressure Infusion Device and Conventional Clear-Cuff Pressure Infuser Bag

| **Feature** | **Homemade Portable Sealed Positive Pressure Infusion Device** | **Clear-Cuff pressure infuser bag** |
| --- | --- | --- |
| **Weight** | ~0.98 kg | 0.2~0.5 kg |
| **Power Requirements** | None (compressed air cylinder) | None (manual squeezing) |
| **Pressure Stability** | Stable and constant (0–40 kPa adjustable; automatic maintenance via pressure regulation) | Variable; requires frequent manual adjustment to maintain pressure |
| **Personnel Needs** | Minimal (simple 4-step operation; automatic shut off) | Higher (requires ongoing manual squeezing and monitoring) |
| **Position Independence** | Gravity-independent; no restrictions on patient position or orientation | Position-dependent; typically requires vertical orientation for optimal flow and stability |
| **Portability/Adaptability** | High (wearable, sealed design suitable for austere environments like battlefields or disasters) | Moderate (portable but less adaptable in dynamic or turbulent conditions) |
