# Supplementary figures and images for: Application of a portable sealed positive pressure infusion device in a porcine model of hemorrhagic shock
Source: Front Med (Lausanne). 2026 Jan 15;13:1738724. doi: 10.3389/fmed.2026.1738724 (PMC12852321; doi:10.3389/fmed.2026.1738724)

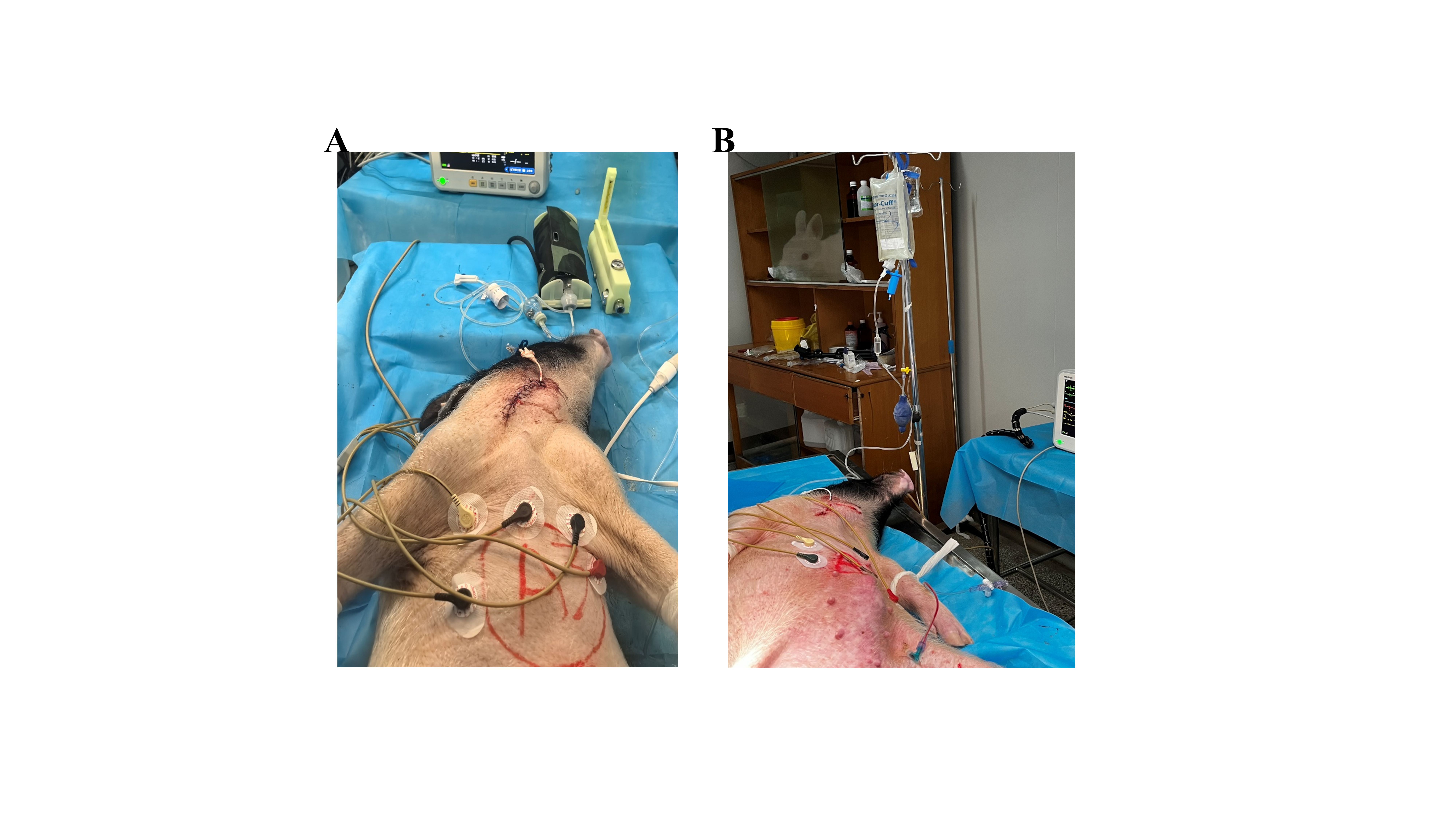

Supplement: Supplementary file 3 [file Image_1.tif]

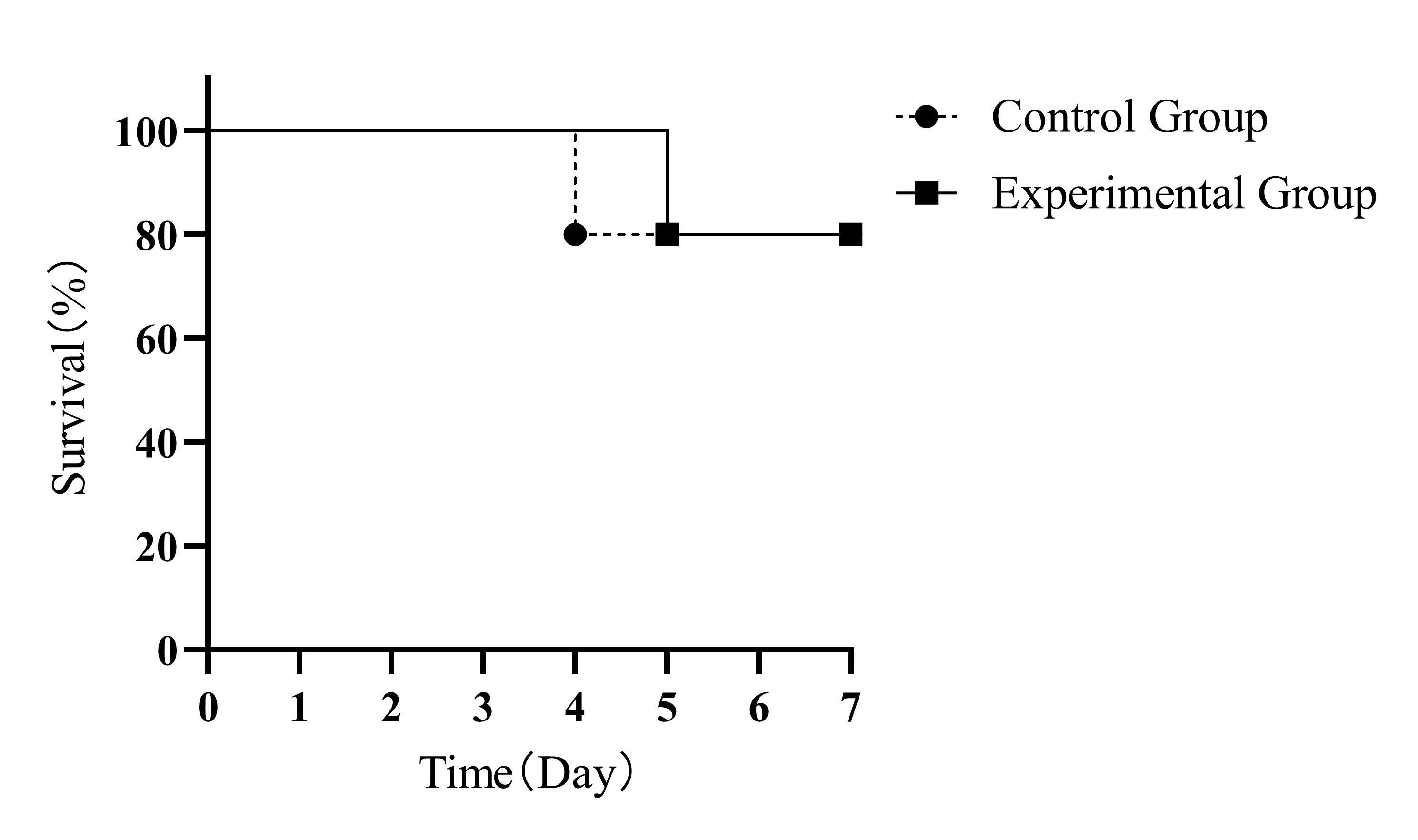

Supplement: Supplementary file 4 [file Image_2.tif]

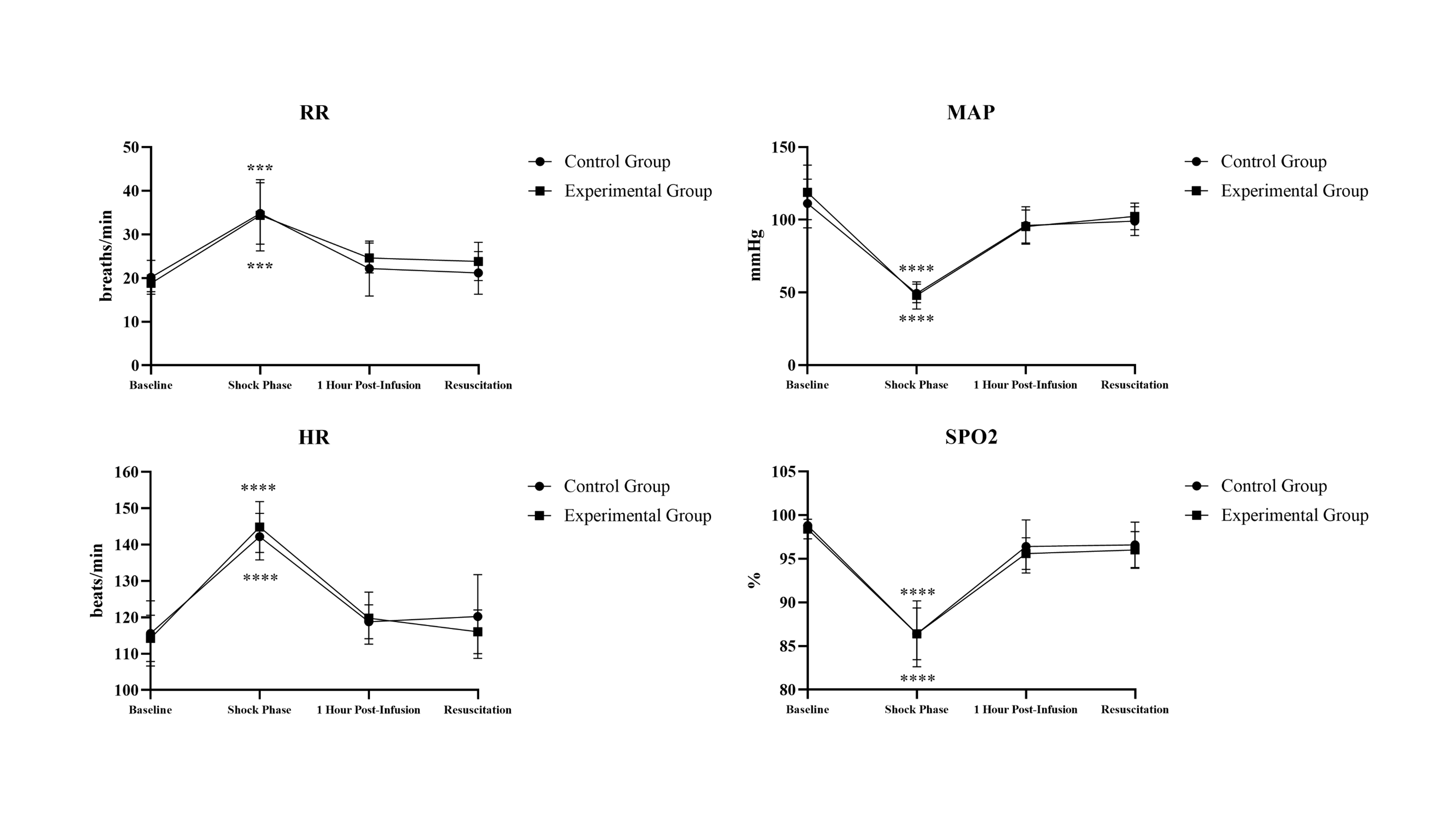

Supplement: Supplementary file 5 [file Image_3.tif]

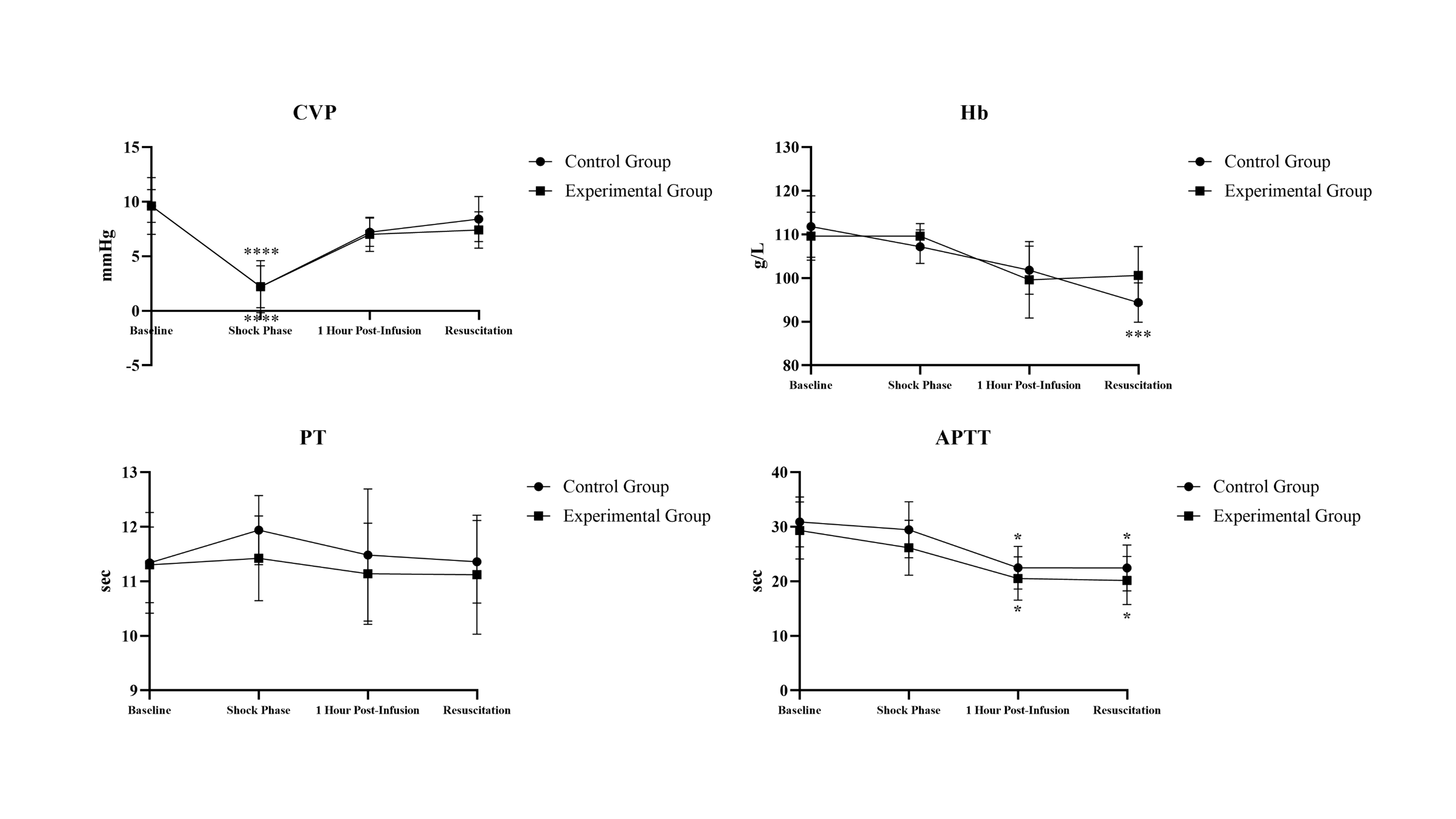

Supplement: Supplementary file 6 [file Image_4.tif]

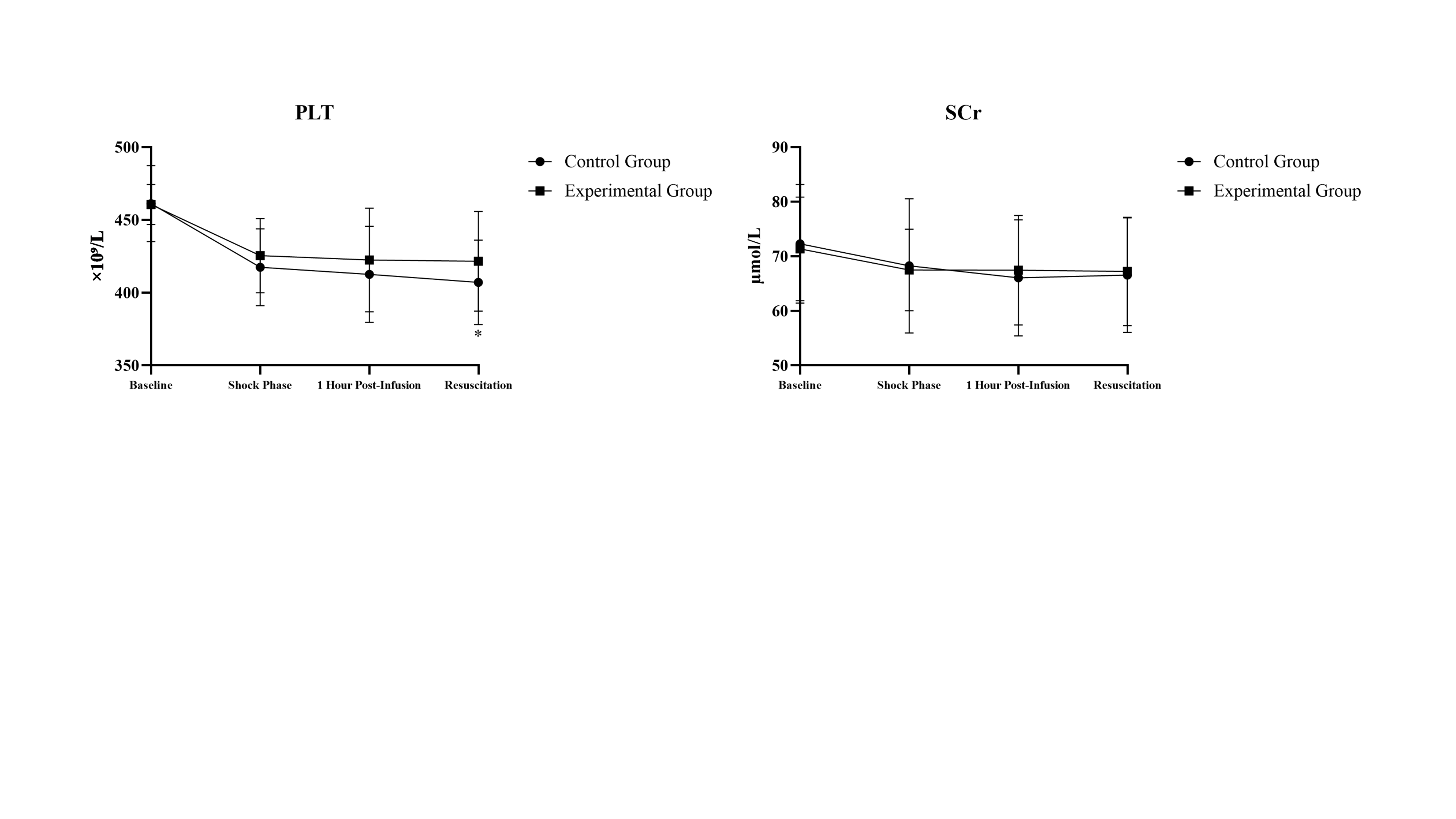

Supplement: Supplementary file 7 [file Image_5.tif]
